# Vesico-Vaginal Fistulæ

**Published:** 1873-04-15

**Authors:** Mary H. Thompson

**Affiliations:** Physician to Hospital for Women and Children, of Chicago


### VESICO-VAGINAL FISTULÆ.—A RARE CASE.

BY DR. MARY H. THOMPSON, PHYSICIAN TO HOSPITAL FOR WOMEN AND CHILDREN, OF CHICAGO.

Nellie W., aged eight years, born of Irish parents, was brought to the Hospital for Women and Children, of Chicago, July 15, 1871.

The mother said "she had a wound in the bladder." On examination, the external genitals and inside of the thighs were found excoriated and inflamed, with the constant irritation of urine flowing over the parts. There was some considerable vaginitis, and a fistulæ large enough to admit the tip of a finger found in the anterior wall of vagina, in the median line, and about midway from the orifice to the junction of vagina and cervix uteri. The edges were thickened, ulcerated, and covered with the urinary deposits and old epithelial scales.

The wound was made under the following circumstances. Five months previously, in March, while playing upon an outside staircase with other children, she fell to the bottom, where some kindling-wood lay scattered. She screamed as though seriously injured. Upon telling where she was hurt a splinter of wood was found between the thighs, where it had penetrated the parts upward and forward. It was withdrawn with considerable force, and found covered with blood. In a day or two the mother found that water was dribbling away without the control of the child.

An attempt was made to heal the parts, and bring fistulæ together by means of applications of the solution of argent nit., twenty grains, aquæ one ounce, applied every third day. The vagina was washed with a solution of sodæ biboras, of the strength of five grains of the salt to the ounce of water, twice daily. The external parts were made well by simple cleanliness.

She continued to improve, and the fistulæ grew smaller until September 27th, when the mother said "she was lonesome, and the child must go home," although we felt sure of success.

She applied for admission to the hospital again, while it was in the barracks, after the fire of October 9, 1871, but was refused on account of the mother.

January 28th, 1873, she presented herself again, when Prof. W. H. Byford determined to operate in the usual way, as the parts were found in a healthy condition.

February 3d, she was anæsthetized and operated upon before the class of the Women's Hospital Medical College of that term. Dr. Sims' speculum was used, as usual. The entire edge of the fistulæ was removed with one cutting of the scissors, and the edges of the fistulæ were brought together with three silver wire sutures placed one above the other, about one-fifth of an inch apart. After the operation there was an attempt made to keep in the urethra a small female catheter, as we had none of sigmoid shape, but it was given up and the child was let to get up and evacuate the bladder every second hour.

No preparation was made for the operation except the giving of eight drops of tincture of ferri mur. three times daily, and of the usual cathartic to clear the intestinal canal of fæcal matter.

After the operation one-eighth grain morph. sulph. was given three times daily. The child was up a part of the day for a week, and all the time after the first week. The sutures were removed, by Prof. Byford, the fifteenth day after the operation, when he found it a perfect success ; and she was discharged *well*, February 23d.

